# Editorial: Emerging microbiological processes and tools that shine in pilot- and field-scale environmental engineering applications

**DOI:** 10.3389/fmicb.2023.1194772

**Published:** 2023-04-13

**Authors:** Yu Miao, Fabrizio Colosimo, Paula J. Mouser, Susan De Long, Andrea Hanson Rhoades

**Affiliations:** ^1^Department of Civil and Environmental Engineering, Northeastern University, Boston, MA, United States; ^2^Department of Marine and Environmental Sciences, Northeastern University, Boston, MA, United States; ^3^Application and Development Department, New England Biolabs, Ipswich, MA, United States; ^4^Department of Civil and Environmental Engineering, University of New Hampshire, Durham, NH, United States; ^5^Department of Civil and Environmental Engineering, Colorado State University, Fort Collins, CO, United States

**Keywords:** biotechnology, multi-omics, microbiomes, emerging contaminant, biotransformation, microbial ecology, biomarker

Microbes play a vital role in countless natural transformation processes that can be harnessed for diverse applications in environmental engineering. Some of the key areas where microbiotechnology benefits environmental engineering include bioremediation, biological wastewater treatment, biosolids treatment, biogas production, and carbon capture and transformation. Due to uncertain climate change–associated disturbance, increasing anthropogenic activities, and poor resource management, global ecosystem services and all therein are stressed. Consequently, there is a need to enhance microbiotechnologies, from the effort of scientists and engineers, for better addressing environmental, ecological, and societal challenges.

Scientists and engineers alike are only beginning to fully understand and subsequently apply microbiotechnologies as environmental solutions at pilot- and field-scales. As such, the overarching goal of this special Research Topic is to integrate perspectives from practicing engineers and professional researchers to help improve the roadmap for translating emerging microbiological, molecular, and mechanistic knowledge obtained from advanced microbial tools into engineering principles and applications to guide the advancement of microbiotechnologies at larger scales. Within this topic, five articles are accepted that highlight studies seeking to discover, characterize, elucidate, and quantify microbial processes in natural or engineered systems. These manuscripts also improve our capacity to understand, model, and predict microbial phenomena, thereby positively impacting environmental solutions.

Metabolomics has emerged as a powerful technique to monitor and quantify microbial processes and products in biological systems with increasing complexity. May et al. utilized ultra-high performance liquid chromatography–high-resolution mass spectrometry (UPHLC–HRMS) to discover and monitor biomarkers related to the metabolome of the SDC-9^TM^ bioaugmentation consortium converting cis-1,2-dichloroethene to vinyl chloride and non-toxic ethene. Nearly 10,000 spectral features were detected, 18 of which were statistically correlated with dechlorination activity. Furthermore, this metabolomics approach importantly indicated that the dechlorination metabolic process did not inhibit the overall functionality within the consortium. The present study illustrates the potential of metabolomics as either a complementary or stand-alone molecular biological tool for monitoring and tracking groundwater bioremediation at sites contaminated by chlorinated ethenes. While further higher resolution analysis is needed to annotate all identified metabolic features, the knowledge and framework established in this study are a crucial first step for advancing field-scale bioremediation metabolomic applications.

Electron shuttles serve as an important means to facilitate direct interspecies electron transfer, particularly for biodegradation applications including anaerobic biological treatment systems. In the review by Deng et al., the authors summarize how a broad range of molecules and materials can serve as electron shuttles, including cytochrome C, riboflavin, conductive nanowires, humic substances, phenazines, carbon-based materials (e.g., granular activated carbon, biochar, nanomaterials), and metal-based materials (e.g., zero valent iron, Fe_2_O_3_ and AQDS). The authors highlight the important but underappreciated role electron shuttles may plan in enhancing anaerobic treatment of antibiotic resistant genes (ARGs). Electron shuttles seem to modulate the microbial community in damaging the cellular structure of the ARG host, or deactivating its mobile genetic elements in order to weaken horizontal gene transfer of ARGs. The authors summarize the potential environmental toxicology and ecological risks, cost-effectiveness for large scale application, and performance for multi-pollutants environments as future research development directions. Recently, carbon-based and meta-based, or carbon-metal composite electron shuttles are being more widely used by researchers and engineers in wastewater treatment and organic solid waste treatment, targeting on antibiotics and ARGs.

Within the field of groundwater remediation, cleanup strategies and goals can be effectively achieved by leveraging the capabilities of microorganisms to biotransform contaminants into lesser or non-toxic end products. However, the performance of bioremediation is limited by uncertain and complex environment and inconsistent monitoring and tracking. Key et al. proposed a systematic and standardized framework for the application of molecular biological tools (MBTs) consisting of three stages that parallel traditional water quality monitoring tools: assessment, design, and performance monitoring, with a multiple lines of evidence (MLOE) approach for data generation and interpretation. Field-scale MBTs data may be generated across subsurface gradients to develop or refine site-specific biogeochemical conceptual models, and the design stage should be guided by the MLOE datasets in determining potential site-specific remedial action. The performance monitoring stage focuses on geochemical parameters and MBTs to evaluate and improve the understanding of remedy progress. Overall, the proposed data-driven framework addresses the broader subsurface contaminant fate and seeks reduce the uncertainty related to biogeochemical processes, thereby increasing the likelihood of the successful implementation of bioremediation.

Within the framework presented by Key et al., Madison et al. informed the design of an enhanced bioremediation approach for a site with trichloroethene (TCE)-impacted groundwater. Following the initial baseline geochemical and microbial characterization, a biostimulation approach to enhance intrinsic bioremediation was implemented by injecting sodium lactate four times in three injection wells that targeted the shallow and intermediate portion of the plume nearest the source area. TCE concentrations decreased following the injections; however, bioremediation stalled with elevated concentrations of cDCE. Interestingly, the stall was due to competition between *Dehalococcoides* and native microorganisms for growth substrates. Consequently, SDC-9^TM^ (comprising more than 10^7^ *Dehalococcoides* cells/mL) was augmented during the fourth injection event, and cDCE concentrations decreased while the concentration of ethene increased. Performance monitoring indicated that natural attenuation was sufficient to meet the remedial and regulatory objectives; thus the site was transitioned to a long-term monitoring regulatory program. This study demonstrated that the application of tools within microbiotechnology in concert with traditional environmental analyses has the potential to reduce site uncertainties, improve bioremediation effectiveness, and provide site managers and stakeholders greater confidence in contaminated site management.

Microbiotechnology is also highly useful in cross-disciplinary applications. Yin et al. utilized 16S rRNA gene sequencing combined with Phylogenetic investigation of Communities by Reconstruction of Unobserved States (PICRUSt) to estimate microbial community function in order to investigate the impact of nitrogenous disinfection byproducts (DBPs) gut flora, and also looked at impacts on mouse intestinal tracts through enzyme activity assays. Three halonitromethanes were investigated, with data revealing oxidative stress and inflammation in mouse small intestine as indicated by a decrease in communities associated with superoxide dismutase and glutathione peroxidase genes. Further, the overall microbial diversity was reduced, and different halonitromethanes led to higher abundance of distinct flora under each condition. The altered intestinal flora demonstrated the potential dysbiosis and damage and disrupted the immune function, reflected by oxidative stress and inflammatory response. Thus, the application of microbiotechnology at large scales can be an emergent tool for human health and disease diagnostic and prevention in the greater context of environmental stewardship, and can lead to detection of subtle, sub-acute toxic impacts, and support more informed regulation of DBPs.

In conclusion, the development and utilization of emergent microbiotechnologies across many disciplines are being recognized by academic and practitioners alike, at application scales ranging from pilot- to field. Potential future directions encompass bioremediation or treatment technology evolution and broader applicability at varying scales. The combination of traditional microbial methods, meta-omics (e.g., genomics, transcriptomics, metabolomics, and proteomics), and bioinformatics blended with big data statistics and machine learning will play a role in advancing our understanding of environmental engineering applications with the primary goal of developing novel solutions.

## Author contributions

AH, YM, and FC organized the Research Topic. SD and PM served as senior editors. YM and AH drafted the editorial manuscript. SD, PM, and FC revised it. All authors handled submissions and discussed and finalized the manuscript.

